# Carotid intima-media thickness, hypertension, and dyslipidemia in obese adolescents

**DOI:** 10.11604/pamj.2019.34.134.18309

**Published:** 2019-11-07

**Authors:** Nur Aisiyah Widjaja, Roedi Irawan, Rendi Aji Prihaningtyas, Meity Ardiana, Meta Herdiana Hanindita

**Affiliations:** 1 Child Health Department, Faculty of Medicine Universitas Airlangga, Dr. Soetomo General Hospital, Surabaya, Indonesia; 2 Medical Doctoral Program Student, Faculty of Medicine Universitas Airlangga, Dr. Soetomo General Hospital, Surabaya, Indonesia; 3 Cardiology and Vascular Medicine Department, Faculty of Medicine Universitas Airlangga, Dr. Soetomo General Hospital, Surabaya, Indonesia

**Keywords:** Obesity, adolescents, CIMT, dyslipidemia, hypertension

## Abstract

**Introduction:**

Obesity is a global health problem with growing prevalence in developing countries. Obesity causes chronic inflammation due to imbalances between pro- and anti-inflammatory cytokines. This causes metabolic complications such as dyslipidemia, hypertension, and cardiovascular disorder. Carotid intima–media thickness (CIMT) is a predictor of atherosclerosis which could be measured easily and non-invasively. Early detection of cardiovascular diseases in obese adolescents at risk is hoped to improve outcomes.

**Methods:**

This is a cross-sectional study on obese adolescents aged 13-16 year old at Pediatric Clinic of Dr. Soetomo General Hospital. Obesity is defined as Body mass index higher than 95^th^ percentiles according to CDC (2000). Dyslipidemia is diagnosed when either an increase in cholesterol, LDL, triglyceride or a decrease in HDL level is found, as recommended by NCPE and American Academy of Pediatrics. Hypertension is defined as an increase of blood pressure > P95 according to age and gender. The differences of CIMT based on dyslipidemia, hypertension, and gender were analyzed with Wilcoxon Mann Whitney with significant p value (p < 0,005).

**Results:**

This study included 59 obese adolescents, consisting of 32 (54.2%) male adolescents and 35 (59.3%) female adolescents. Dyslipidemia was found on 38 (64.4%) adolescents and hypertension was found on 35 (59.3%) adolescents. No difference of CIMT was found between obese adolescents with and without dyslipidemia and with and without hypertension based on gender (p > 0.05).

**Conclusion:**

No difference of CIMT based on gender between adolescents aged below 18. The high number of dyslipidemia and hypertension in obese adolescents need an early detection of cardiovascular complication.

## Introduction

Obesity is a global problem which increases the risk of early death. Obesity causes chronic inflammation through an increase in the production of pro-inflammatory cytokines, which causes metabolic diseases [1]. Adolescents who have risk factors such as obesity, dyslipidemia, hypertension, and diabetes mellitus have higher risk of cardiovascular diseases as adults [2]. Metabolic syndrome increases mortality risk up to 1.5 times [3]. The process of atherosclerosis starts early in obese children and adolescents. Carotid intima media thickness (CIMT) is a subclinical marker of atherosclerosis [2]. Measurement of CIMT is a modality that could be used to assess cardiovascular risk factors non-invasively and has been done since 1980s. Obese adolescents with cardiovascular risk factors have higher CIMT [4]. Majority of study in CIMT as a risk factor of cardiovascular disease are performed in adult and developed country. In developing country, there is a limited study in CIMT of obese adolescents. The aim of this study is to analyze the difference of CIMT between obese adolescents with and without dyslipidemia and with and without hypertension based on gender.

## Methods

A cross-sectional study was done on obese adolescents aged 13-16 year old at Pediatric Clinic Dr. Soetomo General Hospital, Surabaya, Indonesia. Subjects who had Body Mass Index higher than 95^th^ percentile based on BMI presentile in CDC curve according to age and sex were included in this study. Subjects who had consumed corticosteroids within 6 months before study, underwent hormonal therapy or consumed dyslipidemia drugs within 3 months before study, smoked, consumed alcohol, or had endocrine disorder were excluded.

Anthropometry measurement, including body weight and height, was done by trained health workers. Body weight was measured without footwear, accessories, and with clothes that weighed less than 0.1kg using digital scale (Seca, Germany). Body height was measured without footwear or headwear in erect position using stadiometer (Seca, Germany). Body mass index (BMI) was calculated with the formula of body weight (kg) divided by squared body height (meter). Obesity is defined as BMI higher that 95^th^ percentile according to age and gender based on CDC curve (2000). Blood pressure was measured in sitting position after the subject had rested for 10 minutes. Hypertension is defined as blood pressure higher than 95^th^ percentile according to age and gender.

CIMT measurement was done using high-resolution B-mode ultrasonography (Toshiba, Japan) by a cardiologist. Subjects were examined in supine position, with neck minimally extended and probe placed in anterolateral position. Imaging was done on the left common carotid artery. Lipid profile test was done using ELISA method. Triglyceride test was done using Autosera S TG-N Kit (Sekisui Medical Co., Ltd., Japan). LDL, HDL, and total cholesterol tests were done using *Cholestest^®^LDL*, *Cholestest^®^N HDL*, *dan Pureauto^®^S CHO-N* (Sekisui Medical Co., Ltd., Japan). Dyslipidemia is diagnosed when either an increase in cholesterol, LDL, triglyceride or a decrease in HDL level is found, as recommended by NCPE and American Academy of Pediatrics.

Statistic methods: quantitative variables are described in mean and standard deviation. CIMT differences based on dyslipidemia, hypertension, and gender were analyzed using Wilcoxon Mann Whitney with significant p of < 0.05. Analysis was done using SPSS. This study was approved by Ethical Committee in health research of Dr. Soetomo General Hospital (ref. No. 0698/KEPK/X/2018). Parents of the subjects were provided with an informed consent before study. All data obtained from the subject were anonymized.

## Results

This study included 59 obese adolescents, consisting of 32 (54.2%) male adolescents and 35 (59.3%) female adolescents. Dyslipidemia was found on 38 (64.4%) adolescents and hypertension was found on 35 (59.3%) adolescents. Characteristics of the subjects are shown in Table 1. There was no difference in CIMT between female and male adolescents (mean = 0.51 ± 0.12 vs 0.51 ± 0.07; p = 0.50). There was also no difference in CMIT between obese adolescents with or without dyslipidemia (female p = 0.974; male p = 0.313) and with or without hypertension (female p = 0.321; male p = 0.833) based on gender (Table 2).

**Table 1:** Characteristics of the study's subjects

Variable	Mean ± SD
Body weight (kg)	80.77 ± 13.35
Body height (cm)	158.76 ± 7.12
Body mass index (kg/m^2^)	31.99 ± 3.67
CIMT (mm)	0.51 ± 0.10

**Table 2:** Correlation between variables

Variable	Sex	N	CIMT	p
Hypertension	Male	20	0.53 ± 0.08	0.833
	Female	15	0.53 ± 0.14	0.321
Dyslipidemia	Male	24	0.53 ± 0.79	0.313
	Female	14	0.56 ± 0.14	0.974

SD= Standard Deviation

## Discussion

Obesity is related to inflammation as a result of imbalances between pro- and anti-inflammatory cytokines [5]. Inflammation in obesity is marked by an increase in TNF and hsCRP [6]. Storage of abdominal adipose tissue causes cell dysfunction and cardiometabolic diseases in adulthood [7, 8]. The higher the body fat percentage, the higher the risk of cardiovascular diseases [9]. Atherosclerosis proccess starts early among children and adolescents with obesity. CIMT is a subclinical atherosclerosis marker [2]. Obese adolescents have higher CIMT compared to adolescents with normal BMI [10]. Previous study on healthy subjects showed no difference in CIMT between female and male adolescents, but CIMT tends to increase after 10 years of age [11]. A study on obese adolescents showed no difference in CIMT between males and females, but an association between CIMT and arterial stiffness was found, especially among female adolescents [12]. Results from this study is in accordance with that study. When a child turns 10 year old, puberty starts and hormonal change could cause changes in body fat composition [13].

In this study, majority of subject had dyslipidemia and hypertension (Table 1). CIMT increases when dyslipidemia, hypertension, and diabetes mellitus is present [14]. Obese adolescents have a 6.5 times higher risk of pre-hypertension or hypertension [15]. Blood pressure has direct effect on CIMT [4]. Hypertension causes hypertrophy of the tunica media of blood vessels, therefore increasing CIMT [16]. However, another study on children and adolescents showed that there is no difference in CIMT between subjects with obesity and with metabolic syndromes [2]. This study did not find a difference in CIMT between subjects with and without hypertension based on gender. Previous study mentioned that CIMT is associated with age, but not blood pressure [10]. No difference in CIMT between subjects with and without dyslipidemia based on gender was found (Table 2). Previous study reported that obesity could affect BMI if cardiovascular risk factor, such as dyslipidemia, is present [4]. High level of triglyceride is associated with increased CIMT [2]. Gender does not affect the wall of normal common carotid artery until the age of 18 with progressive thickening of the blood vessel's wall [17].

There were some limitation in this study. First, there was a limited subject in this study. Second, the low sensitivity of high-resolution B-mode ultrasonography could affect CIMT measurement and might not be able to detect small differences, as a previous study [11]. There was still a limited study in CIMT of obese adolescence in developing country. Detecting of early risk of cardiovascular disease in obese adolescents are needed. Further studies with a greater number of subject and control subject who has a normal BMI are needed to assess the risk of cardiovascular disease using CIMT in obese adolescents.

## Conclusion

No difference in CIMT was found between obese adolescents with and without hypertension and with and without dyslipidemia based on gender. Age below 18 does not affect CIMT thickness may due to unstarted progressive thickening of the wall of the common carotid artery. There was a higher number of dyslipidemia and hypertension in obese adolescents. The further study with a greater number of subject and control subject are needed to assess the risk of cardiovascular disease in obese adolescents.

### What is known about this topic

- No difference in CIMT was found between obese adolescents based on gender;
- Obese adolescents can suffered from hypertension and dyslipidemia;
- Hyperension and dyslipidemia can influence the CIMT.

### What this study adds

- No difference in CIMT was found between obese adolescents with and without hypertension and with and without dyslipidemia based on gender;
- Unstarted progressive thickening of the wall of the common carotid artery at the age below 18 can cause no differences in CIMT;
- Prevalence of dyslipidemia and hypertension in obese adolescents are high in developing country, such as Indonesia.

## Competing interests

The authors declare no competing interests.
